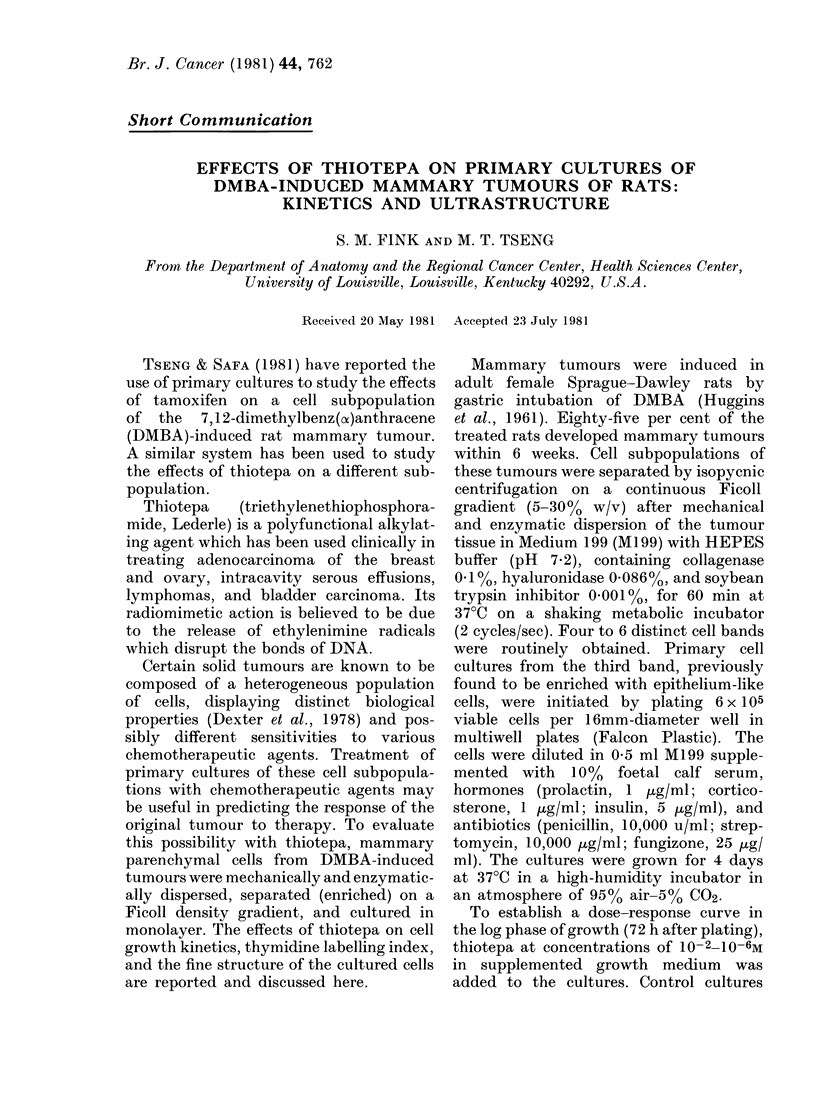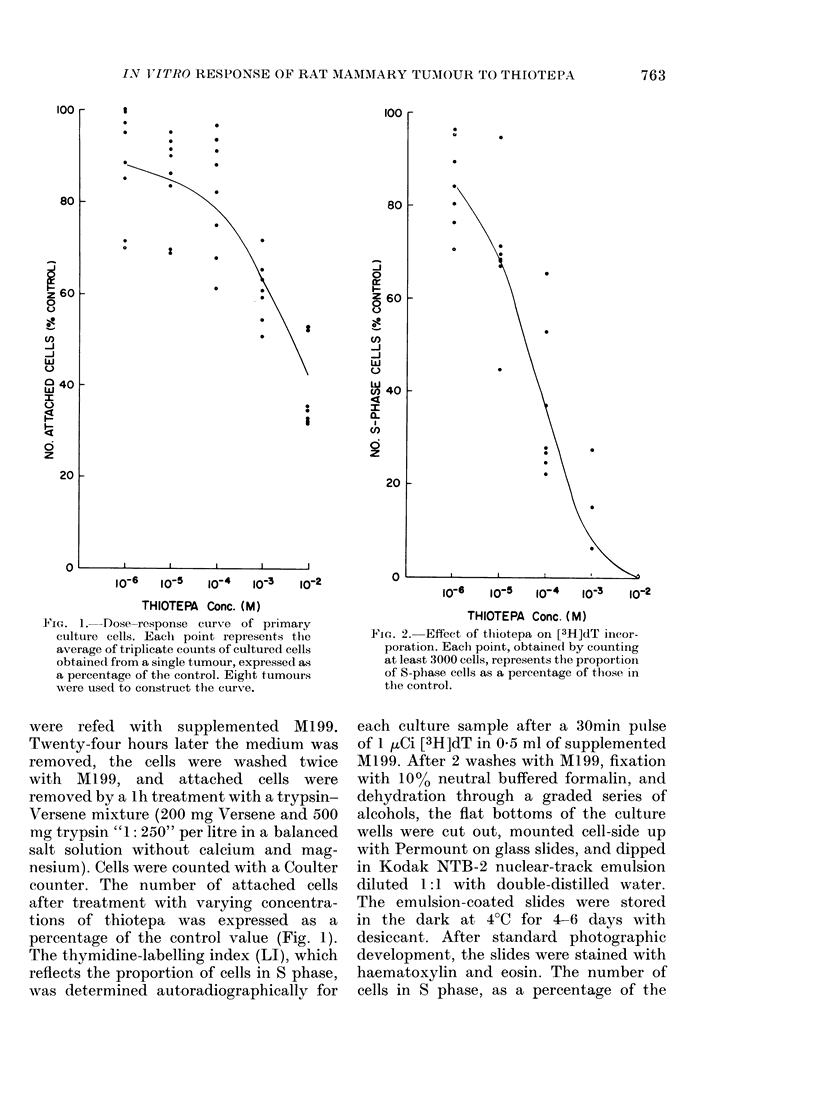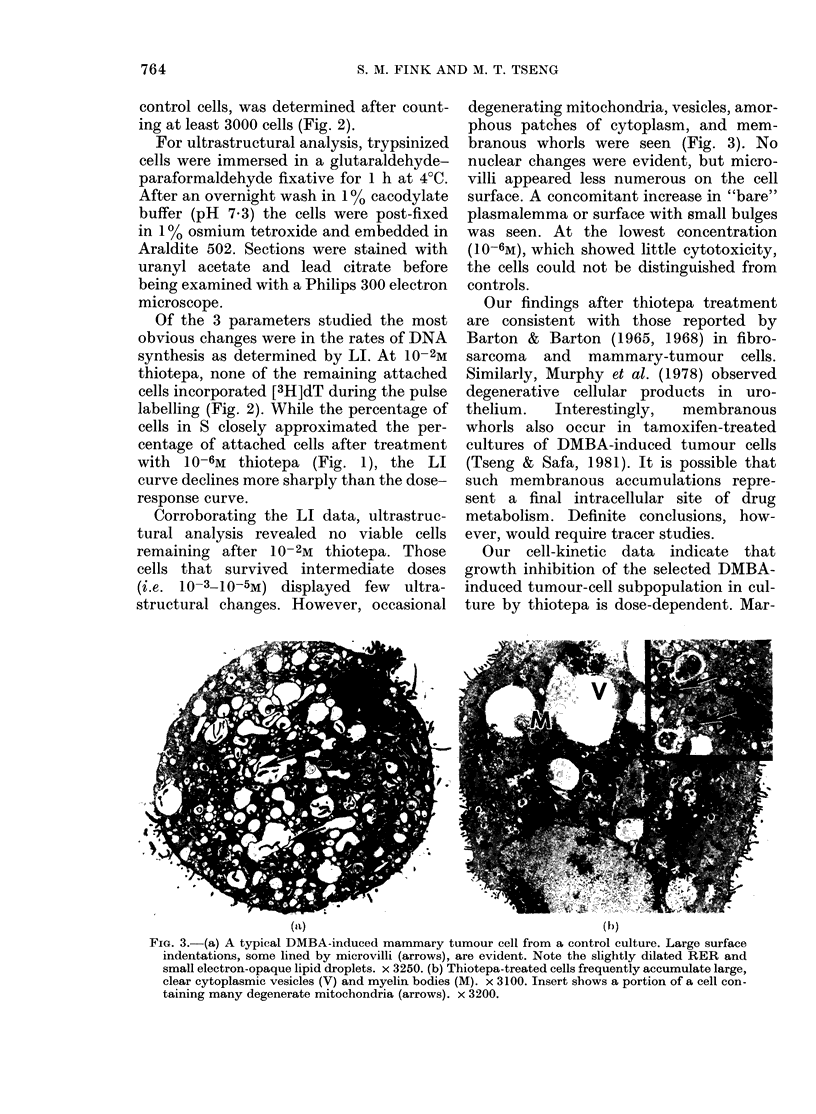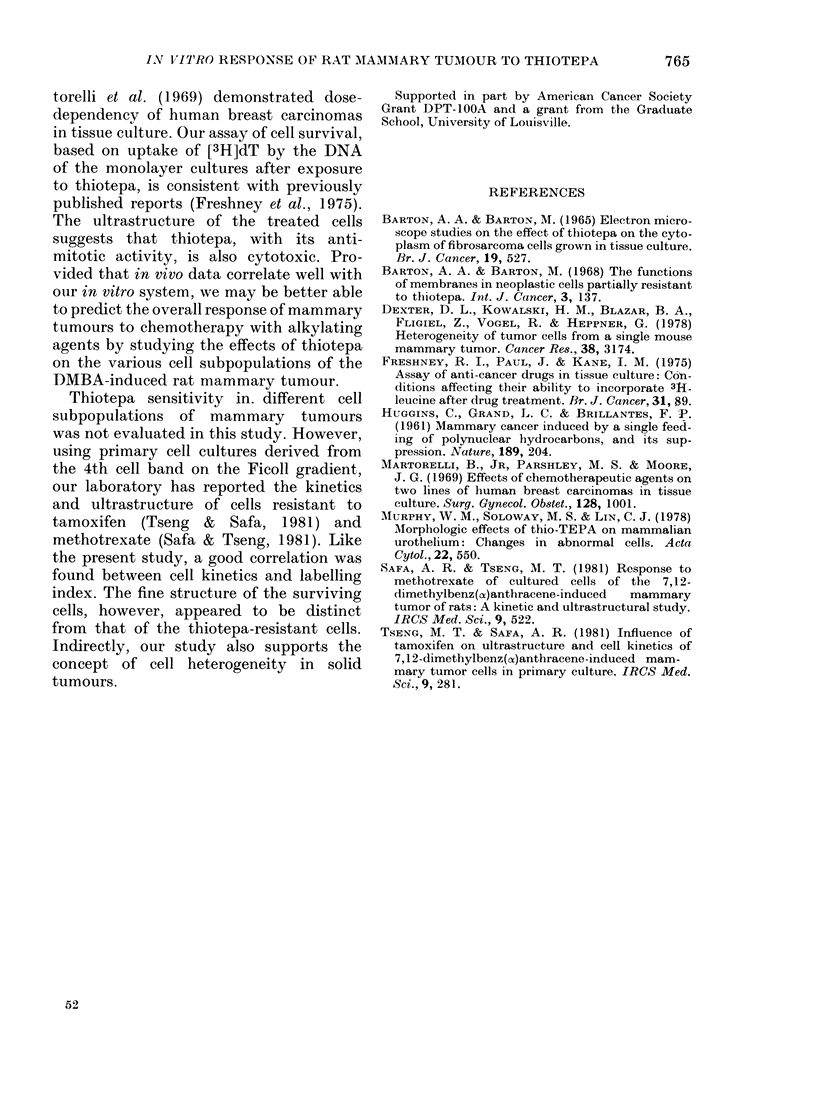# Effects of thiotepa on primary cultures of DMBA-induced mammary tumours of rats: kinetics and ultrastructure.

**DOI:** 10.1038/bjc.1981.267

**Published:** 1981-11

**Authors:** S. M. Fink, M. T. Tseng

## Abstract

**Images:**


*Br. J. Cancer* (1981) **44**, 762

***Short Communication***

### EFFECTS OF THIOTEPA ON PRIMARY CULTURES OF DMBA-INDUCED MAMMARY TUMOURS OF RATS: KINETICS AND ULTRASTRUCTURE

S. M. FINK AND M. T. TSENG

*From the Department of Anatomy and the Regional Cancer Center, Health Sciences Center, University of Louisville, Louisville, Kentucky* 40292, *U.S.A.*

Received 20 May 1981 Accepted 23 July 1981

TSENG & SAFA (1981) have reported the use of primary cultures to study the effects of tamoxifen on a cell subpopulation of the 7,12-dimethylbenz(α)anthracene (DMBA)-induced rat mammary tumour. A similar system has been used to study the effects of thiotepa on a different subpopulation.

Thiotepa (triethylenethiophosphoramide, Lederle) is a polyfunctional alkylating agent which has been used clinically in treating adenocarcinoma of the breast and ovary, intracavity serous effusions, lymphomas, and bladder carcinoma. Its radiomimetic action is believed to be due to the release of ethylenimine radicals which disrupt the bonds of DNA.

Certain solid tumours are known to be composed of a heterogeneous population of cells, displaying distinct biological properties (Dexter *et al.*, 1978) and possibly different sensitivities to various chemotherapeutic agents. Treatment of primary cultures of these cell subpopulations with chemotherapeutic agents may be useful in predicting the response of the original tumour to therapy. To evaluate this possibility with thiotepa, mammary parenchymal cells from DMBA-induced tumours were mechanically and enzymatically dispersed, separated (enriched) on a Ficoll density gradient, and cultured in monolayer. The effects of thiotepa on cell growth kinetics, thymidine labelling index, and the fine structure of the cultured cells are reported and discussed here.

Mammary tumours were induced in adult female Sprague–Dawley rats by gastric intubation of DMBA (Huggins *et al.*, 1961). Eighty-five per cent of the treated rats developed mammary tumours within 6 weeks. Cell subpopulations of these tumours were separated by isopycnic centrifugation on a continuous Ficoll gradient (5–30% w/v) after mechanical and enzymatic dispersion of the tumour tissue in Medium 199 (M199) with HEPES buffer (pH 7·2), containing collagenase 0·1%, hyaluronidase 0·086%, and soybean trypsin inhibitor 0·001%, for 60 min at 37°C on a shaking metabolic incubator (2 cycles/sec). Four to 6 distinct cell bands were routinely obtained. Primary cell cultures from the third band, previously found to be enriched with epithelium-like cells, were initiated by plating $6\times10^5$ viable cells per 16mm-diameter well in multiwell plates (Falcon Plastic). The cells were diluted in 0·5 ml M199 supplemented with 10% foetal calf serum, hormones (prolactin, 1 μg/ml; corticosterone, 1 μg/ml; insulin, 5 μg/ml), and antibiotics (penicillin, 10,000 u/ml; streptomycin, 10,000 μg/ml; fungizone, 25 μg/ml). The cultures were grown for 4 days at 37°C in a high-humidity incubator in an atmosphere of 95% air–5% $CO_2$.

To establish a dose–response curve in the log phase of growth (72 h after plating), thiotepa at concentrations of $10^{-2}$–$10^{-6}$M in supplemented growth medium was added to the cultures. Control cultures

FIG. 1.—Dose-response curve of primary culture cells. Each point represents the average of triplicate counts of cultured cells obtained from a single tumour, expressed as a percentage of the control. Eight tumours were used to construct the curve.

FIG. 2.—Effect of thiotepa on [$^3$H]dT incorporation. Each point, obtained by counting at least 3000 cells, represents the proportion of S-phase cells as a percentage of those in the control.

were refed with supplemented M199. Twenty-four hours later the medium was removed, the cells were washed twice with M199, and attached cells were removed by a 1h treatment with a trypsin–Versene mixture (200 mg Versene and 500 mg trypsin "1 : 250" per litre in a balanced salt solution without calcium and magnesium). Cells were counted with a Coulter counter. The number of attached cells after treatment with varying concentrations of thiotepa was expressed as a percentage of the control value (Fig. 1). The thymidine-labelling index (LI), which reflects the proportion of cells in S phase, was determined autoradiographically for each culture sample after a 30min pulse of 1 $\mu$Ci [$^3$H]dT in 0·5 ml of supplemented M199. After 2 washes with M199, fixation with 10% neutral buffered formalin, and dehydration through a graded series of alcohols, the flat bottoms of the culture wells were cut out, mounted cell-side up with Permount on glass slides, and dipped in Kodak NTB-2 nuclear-track emulsion diluted 1:1 with double-distilled water. The emulsion-coated slides were stored in the dark at 4°C for 4–6 days with desiccant. After standard photographic development, the slides were stained with haematoxylin and eosin. The number of cells in S phase, as a percentage of the control cells, was determined after counting at least 3000 cells (Fig. 2).

For ultrastructural analysis, trypsinized cells were immersed in a glutaraldehyde–paraformaldehyde fixative for 1 h at 4°C. After an overnight wash in 1% cacodylate buffer (pH 7·3) the cells were post-fixed in 1% osmium tetroxide and embedded in Araldite 502. Sections were stained with uranyl acetate and lead citrate before being examined with a Philips 300 electron microscope.

Of the 3 parameters studied the most obvious changes were in the rates of DNA synthesis as determined by LI. At $10^{-2}$M thiotepa, none of the remaining attached cells incorporated [$^3$H]dT during the pulse labelling (Fig. 2). While the percentage of cells in S closely approximated the percentage of attached cells after treatment with $10^{-6}$M thiotepa (Fig. 1), the LI curve declines more sharply than the dose–response curve.

Corroborating the LI data, ultrastructural analysis revealed no viable cells remaining after $10^{-2}$M thiotepa. Those cells that survived intermediate doses (*i.e.* $10^{-3}$–$10^{-5}$M) displayed few ultrastructural changes. However, occasional degenerating mitochondria, vesicles, amorphous patches of cytoplasm, and membranous whorls were seen (Fig. 3). No nuclear changes were evident, but microvilli appeared less numerous on the cell surface. A concomitant increase in "bare" plasmalemma or surface with small bulges was seen. At the lowest concentration ($10^{-6}$M), which showed little cytotoxicity, the cells could not be distinguished from controls.

Our findings after thiotepa treatment are consistent with those reported by Barton & Barton (1965, 1968) in fibrosarcoma and mammary-tumour cells. Similarly, Murphy *et al.* (1978) observed degenerative cellular products in urothelium. Interestingly, membranous whorls also occur in tamoxifen-treated cultures of DMBA-induced tumour cells (Tseng & Safa, 1981). It is possible that such membranous accumulations represent a final intracellular site of drug metabolism. Definite conclusions, however, would require tracer studies.

Our cell-kinetic data indicate that growth inhibition of the selected DMBA-induced tumour-cell subpopulation in culture by thiotepa is dose-dependent. Mar-

(a) (b)

FIG. 3.—(a) A typical DMBA-induced mammary tumour cell from a control culture. Large surface indentations, some lined by microvilli (arrows), are evident. Note the slightly dilated RER and small electron-opaque lipid droplets. × 3250. (b) Thiotepa-treated cells frequently accumulate large, clear cytoplasmic vesicles (V) and myelin bodies (M). × 3100. Insert shows a portion of a cell containing many degenerate mitochondria (arrows). × 3200.

torelli *et al.* (1969) demonstrated dose-dependency of human breast carcinomas in tissue culture. Our assay of cell survival, based on uptake of [$^3$H]dT by the DNA of the monolayer cultures after exposure to thiotepa, is consistent with previously published reports (Freshney *et al.*, 1975). The ultrastructure of the treated cells suggests that thiotepa, with its anti-mitotic activity, is also cytotoxic. Provided that *in vivo* data correlate well with our *in vitro* system, we may be better able to predict the overall response of mammary tumours to chemotherapy with alkylating agents by studying the effects of thiotepa on the various cell subpopulations of the DMBA-induced rat mammary tumour.

Thiotepa sensitivity in. different cell subpopulations of mammary tumours was not evaluated in this study. However, using primary cell cultures derived from the 4th cell band on the Ficoll gradient, our laboratory has reported the kinetics and ultrastructure of cells resistant to tamoxifen (Tseng & Safa, 1981) and methotrexate (Safa & Tseng, 1981). Like the present study, a good correlation was found between cell kinetics and labelling index. The fine structure of the surviving cells, however, appeared to be distinct from that of the thiotepa-resistant cells. Indirectly, our study also supports the concept of cell heterogeneity in solid tumours.

Supported in part by American Cancer Society Grant DPT-100A and a grant from the Graduate School, University of Louisville.

REFERENCES

BARTON, A. A. & BARTON, M. (1965) Electron microscope studies on the effect of thiotepa on the cytoplasm of fibrosarcoma cells grown in tissue culture. *Br. J. Cancer*, **19**, 527.

BARTON, A. A. & BARTON, M. (1968) The functions of membranes in neoplastic cells partially resistant to thiotepa. *Int. J. Cancer*, **3**, 137.

DEXTER, D. L., KOWALSKI, H. M., BLAZAR, B. A., FLIGIEL, Z., VOGEL, R. & HEPPNER, G. (1978) Heterogeneity of tumor cells from a single mouse mammary tumor. *Cancer Res.*, **38**, 3174.

FRESHNEY, R. I., PAUL, J. & KANE, I. M. (1975) Assay of anti-cancer drugs in tissue culture: Conditions affecting their ability to incorporate $^3$H-leucine after drug treatment. *Br. J. Cancer*, **31**, 89.

HUGGINS, C., GRAND, L. C. & BRILLANTES, F. P. (1961) Mammary cancer induced by a single feeding of polynuclear hydrocarbons, and its suppression. *Nature*, **189**, 204.

MARTORELLI, B., JR, PARSHLEY, M. S. & MOORE, J. G. (1969) Effects of chemotherapeutic agents on two lines of human breast carcinomas in tissue culture. *Surg. Gynecol. Obstet.*, **128**, 1001.

MURPHY, W. M., SOLOWAY, M. S. & LIN, C. J. (1978) Morphologic effects of thio-TEPA on mammalian urothelium: Changes in abnormal cells. *Acta Cytol.*, **22**, 550.

SAFA, A. R. & TSENG, M. T. (1981) Response to methotrexate of cultured cells of the 7,12-dimethylbenz(α)anthracene-induced mammary tumor of rats: A kinetic and ultrastructural study. *IRCS Med. Sci.*, **9**, 522.

TSENG, M. T. & SAFA, A. R. (1981) Influence of tamoxifen on ultrastructure and cell kinetics of 7,12-dimethylbenz(α)anthracene-induced mammary tumor cells in primary culture. *IRCS Med. Sci.*, **9**, 281.